# Information transmission efficiency in neuronal communication systems

**DOI:** 10.1186/1471-2202-14-S1-P217

**Published:** 2013-07-08

**Authors:** Bartosz Paprocki, Janusz Szczepanski, Dorota Kolbuk

**Affiliations:** 1 Institute of Mechanics and Applied Computer Sciences, Kazimierz Wielki University, Bydgoszcz, Poland; 2 Institute of Fundamental Technological Research, Polish Academy of Sciences, Warsaw, Poland

## 

**Figure 1:**
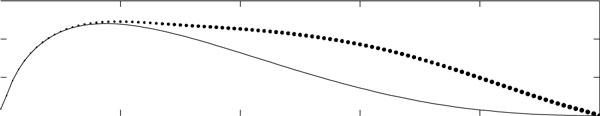
**Mutual information dependency on *synaptic success, s*, in single-layer neural network**. Maximal mutual information values (dotted line) and these achieved at *s *= 1 (solid). Size of a given dot is proportional to 1−*s*, indicating the bigger the dot, the corresponding mutual information value is achieved at lower *s *[3].

The nature and efficiency of brain transmission processes, its high reliability and efficiency is one of the most elusive area of contemporary science [1]. We study information transmission efficiency by considering a neuronal communication as a Shannon-type channel. Thus, using high quality entropy estimators, we evaluate the mutual information between input and output signals. We assume model of neuron proposed by Levy and Baxter [2], which incorporates all essential qualitative mechanisms participating in neural transmission process. We analyze how the *synaptic failure, activation threshold *and characteristics of the input source affect the efficiency. Two types of network architectures are considered. We start by a single-layer feedforward network and next we study *brain-like *networks which contains components such as *excitatory *and *inhibitory *neurons or *long-range connections*. It turned out that, especially for lower activation thresholds, significant synaptic noise can lead even to twofold [Figure 1] increase of the transmission efficiency [3]. Moreover, the more amplifying the amplitude fluctuation is, the more positive is the role of synaptic noise [4]. Our research also shows that all brain-like network components, in broad range of conditions, significantly improve the information-energetic efficiency. It turned out that inhibitory neurons can improve the information-energetic transmission efficiency by 50 percent, while long-range connections can improve the efficiency even by 70 percent. The knowledge of the effects of the long-range connections could be particulary useful when we consider possible reconstruction or support of them applying biomaterials [5,6]. We also showed that the most effective is the network with the smallest size: we found that two times increase of the size can cause even three times decrease of the information-energetic efficiency [7].
